# PM_2.5_ Pollution and Inhibitory Effects on Industry Development: A Bidirectional Correlation Effect Mechanism

**DOI:** 10.3390/ijerph16071159

**Published:** 2019-03-31

**Authors:** Jibo Chen, Keyao Chen, Guizhi Wang, Lingyan Wu, Xiaodong Liu, Guo Wei

**Affiliations:** 1 School of Mathematics and Statistics, Nanjing University of Information Science & Technology, Nanjing 210044, China; chenjibo@nuist.edu.cn (J.C.); wgz@nuist.edu.cn (G.W.); wulingyan14@126.com (L.W.); 2 National Climate Center, China Meteorological Administration, Beijing 100081, China; 3 School of Computing, Edinburgh Napier University, Edinburgh EH10 5DT, UK; x.liu@napier.ac.uk; 4 Department of Mathematics and Computer Science, University of North Carolina at Pembroke, Pembroke, NC 28372, USA; guo.wei@uncp.edu

**Keywords:** haze pollution, vector autoregression model, impulse response function, variance decomposition, industry development

## Abstract

In this paper, a vector autoregression (VAR) model has been constructed in order to analyze a two-way mechanism between PM_2.5_ pollution and industry development in Beijing via the combination of an impulse response function and variance decomposition. According to the results, long-term equilibrium interconnection was found between PM_2.5_ pollution and the development of primary, secondary, and tertiary industries. One-way Granger causalities were found in the three types of industries shown to contribute to PM_2.5_ pollution, though the three industries showed different scales of influences on the PM_2.5_ pollution that varied for about 1–2 years. The development of the primary and secondary industries increased the emission of PM_2.5_, but the tertiary industry had an inhibitory effect. In addition, PM_2.5_ pollution had a certain inhibitory effect on the development of the primary and secondary industries, but the inhibition of the tertiary industry was not significant. Therefore, the development of the tertiary industry can contribute the most to the reduction of PM_2.5_ pollution. Based on these findings, policy-making recommendations can be proposed regarding upcoming pollution prevention strategies.

## 1. Introduction

Haze pollution is a serious problem in China. For example, in 12–17 January 2018, Beijing and its surrounding areas suffered from severe regional heavy haze pollution, where the highest concentrations of PM_2.5_ reached over 900 μg/m^3^ [1]. Numerous studies in China have revealed that there is mutual influence between industry development (mostly in the form of economic growth) and haze pollution; however, particular relationships between different industry sectors and haze pollution differs. The classification of different industry sectors is defined by the National Bureau of Statistics of China, which divides industry into primary, secondary, and tertiary industry according to their economic activities [2]. The primary industry refers to agriculture, forestry, animal husbandry, and fishery (excluding relevant services, which belongs to the tertiary industry). The secondary industry refers to the mining industry (excluding mining assistance services), manufacturing (excluding metal-related production services, machinery, and equipment repair services, etc.), energy production and supply (e.g., electricity, heat, gas, and water), and architecture, engineering, and construction. The tertiary industry refers to those that are not classified into the primary and secondary industry, such as transportation, information, finance, commerce, catering, education, public services, and other non-material production sectors [3].

Stubble burning in the primary industry can release a lot of persistent pollutants in the air [4] and, therefore, cause air pollution with a certain circulation background and ground meteorological characteristics [5]. Air pollution caused by stubble burning has significant characteristics of explosiveness, heavy pollution, short durations, and increased particulate matter concentration [6]. According to stubble burning data from 31 provinces in China during 1997 to 2013, 1.036 million tons of PM_2.5_ have been released per year due to stubble burning [7]. Zhao et al. [8] also suggested that agricultural activities were an important source of haze-pollutant emissions. Meanwhile, haze pollution is taken as one of the major disasters that constitute agricultural production [9]. China’s grain output was reduced by about 3 billion kilograms annually as a result of atmospheric pollution [10]. Dust that covers the surface of leaves would shield 60% of light intensity, resulting in about a 20% reduction in photosynthetic product [11], and eventually lead to yield loss [12,13]. Trace-metals such as lead and mercury in haze can damage cell membranes [14], inhibit mineral absorption [15,16], and inhibit photosynthesis and transpiration as well [17]. Zeng et al. [18] adopted a market value method to estimate agricultural economic losses related to atmospheric pollution in Xi’an in 2013, which was up to 1.985 billion yuan.

In the secondary industry, industrial emissions and construction dust are the main sources of haze pollution. Power trade and metallurgical trade are the two major pollutant emissions from industrial sources in Beijing [19]. An increase in the proportion of industry in social economy would lead to severe haze pollution [20,21,22]. A case study conducted in Beijing showed that construction dust contributed 31.3 μg/m^3^ and 9.6 μg/m^3^ to concentrations of PM_10_ and PM_2.5_, respectively [23]. Another study conducted in Langfang City, Hebei Province, China, concluded that annual emissions of PM_2.5_ and PM_10_ caused by construction dust reached 7546 tons and 19,968 tons in 2014, respectively [24]. In terms of the significance of haze pollution, it can promote the transformation of industrial production methods [25], improve energy efficiency [26], and optimize industry structure [27]. Haze can also reduce the flashover performance of insulators and thereby easily lead to pollution flashover [28,29]. Moreover, reduced visibility caused by haze pollution may threaten the safety of construction, the efficiency of construction operations, and increase the cost of construction [30,31,32,33,34].

In the tertiary industry, transportation is an important contributor to haze pollution [35,36]. Exhaust emissions of motor vehicles are the direct cause of outdoor PM_2.5_ and PM_10_ [37,38,39,40]. Although haze pollution would, to a certain extent, stimulate the output of health services [41], it is far less than the damage it causes. In the tertiary industry, transportation and tourism are the most vulnerable sectors [42] because of reduced visibility and potential health damage [43,44]. In January 2013, direct economic losses in the national transportation industry of China were approximately 76.6 million USD because of the haze pollution [45]. Inbound tourists’ perceptions of China’s environment were generally low because of haze weather [46]. Most importantly, haze pollution has a negative impact on both tourism resources and its transportation [47].

Most of the existing research outcomes focus on the mechanism of haze influences, but rarely do they study the dynamic characteristics between haze pollution and particular industry sectors. Several scholars have tried to study the relationship between environmental pollution and economic development through vector autoregression (VAR) models, panel data models, or other methods [48,49,50,51,52]. However, most of these studies failed to consider industry refinement, and they used per capita gross domestic product (GDP) only as an indicator of economic progress. How to properly investigate, describe, and sculpt the relationship between industry development and haze (PM_2.5_) pollution is a challenging question; therefore, this becomes the main research purpose in this paper. Precisely, by studying the relationship between haze pollution and various industry sectors, a further dynamic interrelation is explored in this paper. Based on Beijing’s PM_2.5_ concentration and the gross outputs from the three major sectors of industry from the first quarter of 2010 to the second quarter of 2017, a VAR model has been established in this paper. By utilizing the impulse response function and variance decomposition, the dynamic interconnection between the PM_2.5_ pollution levels and the development of the three main industry sectors has been analyzed. This study can offer a new perspective for studying the relationship between haze pollution and economic development, and it can provide new ideas for haze pollution management as well.

## 2. Materials and Methods

### 2.1. A Vector Autoregression (VAR) Model

Sims [53] introduced a popular VAR model to econometrics in 1980, and the VAR model has been widely used since then [54,55]. In the VAR model, endogenous variables were used to regress the lag value of each variable in the system for studying the dynamic relationship between these system variables and the endogenous variables. The *p*-order VAR model is denoted as VAR (*p*), which is generally given in (1).

(1) $$Y_{t}=\Pi_{1}Y_{t-1}+\Pi_{2}Y_{t-2}+\cdots +\Pi_{p}Y_{t-p}+HX_{t}+U_{t},\;t=1,\;2,\;\cdots ,\;T,$$

where T is the sample size. $Y_{t}={\left(y_{1,t},\;y_{2,t},\;\cdots ,\;y_{k,t}\right)}^{T}$ is a k dimensional endogenous variable vector. $X_{t}={\left(x_{1,t},\;x_{2,t},\;\cdots ,\;x_{d,t}\right)}^{T}$ is a d dimensional endogenous variable vector. $\Pi_{1},\;\Pi_{2},\;\cdots ,\;\Pi_{p}$ are the unknown k×k parametric matrices. H is a k×d dimensional estimated coefficient matrix. $U_{t}={\left(u_{1,t},\;u_{2,t},\;\cdots ,\;u_{k,t}\right)}^{T}$ is a k dimensional random error vector (i.e., a white noise process). It satisfies:

(2) $$E\left(U_{t}\right)=0,\;E\left(u_{i,t},\;u_{j,t}^{T}\right)=\{\begin{array}{c} \sigma_{ij}\;\;\;i=j\\ 0\;\;\;i\neq j \end{array},$$

where $\Sigma =\sigma_{i}^{2}{Ι}_{k};\;i=1,\;2,\;\cdots ,\;k$; and $\sigma_{i}^{2}=Var\left(u_{i,t}\right)$. ${Ι}_{k}$ is a k-th order identity matrix.

Determining the optimal lag order is especially important for establishing the VAR model. Increasing the lag order appropriately can increase the autocorrelation in the error term, but it also easily affects the degree of freedom of the model. Therefore, a comprehensive method was proposed to determine the lag order of the model, based on the likelihood ratio (LR) test statistic, final prediction error (FPE), Akaike information criterion (AIC), Schwarz information criterion (SC), and Hannan-Quinn (HQ) information criterion.

### 2.2. Checking the Model

#### 2.2.1. Stability Test

The stability is characterized by the variation in the values of the parameters in a given model.

When p=1, the characteristic equation of the VAR model shown in (1) is shown in (3).

(3) $$\left|\Pi_{1}-\lambda I\right|=0.$$

For the VAR model, an equivalent condition for the stability can be defined because the solutions of Equation (3) are within the unit circle.

When p>1, the following equation can be established:

(4) $$Y_{t-1}=Y_{t-1};\;Y_{t-2}=Y_{t-2};\;\;\vdots \;Y_{t-p+1}=Y_{t-p+1}.$$

Combining Equations (3) and (4) can lead to (5).

(5) $${\left(\begin{array}{c} Y_{t}\\ Y_{t-1}\\ Y_{t-2}\\ \vdots \\ Y_{t-p+1} \end{array}\right)}_{kp\times 1}={\left(\begin{array}{c} C\\ 0\\ 0\\ \vdots \\ 0 \end{array}\right)}_{kp\times 1}+{\left(\begin{array}{ccccc} \Pi_{1} & \Pi_{2} & \cdots & \Pi_{p-1} & \Pi_{p}\\ I & 0 & \cdots & 0 & 0\\ 0 & I & \cdots & 0 & 0\\ \vdots & \vdots & \vdots & \vdots & \vdots \\ 0 & 0 & \cdots & I & 0 \end{array}\right)}_{kp\times kp}{\left(\begin{array}{c} Y_{t-1}\\ Y_{t-2}\\ Y_{t-3}\\ \vdots \\ Y_{t-p} \end{array}\right)}_{kp\times 1}+{\left(\begin{array}{c} U_{t}\\ 0\\ 0\\ \vdots \\ 0 \end{array}\right)}_{kp\times 1}.$$

Given:

(6) $$Z_{t}={\left(\begin{array}{c} Y_{t}\\ Y_{t-1}\\ Y_{t-2}\\ \vdots \\ Y_{t-p+1} \end{array}\right)}_{kp\times 1},A={\left(\begin{array}{c} C\\ 0\\ 0\\ \vdots \\ 0 \end{array}\right)}_{kp\times 1},E_{t}={\left(\begin{array}{c} U_{t}\\ 0\\ 0\\ \vdots \\ 0 \end{array}\right)}_{kp\times 1},\Phi ={\left(\begin{array}{ccccc} \Pi_{1} & \Pi_{2} & \cdots & \Pi_{p-1} & \Pi_{p}\\ I & 0 & \cdots & 0 & 0\\ 0 & I & \cdots & 0 & 0\\ \vdots & \vdots & \vdots & \vdots & \vdots \\ 0 & 0 & \cdots & I & 0 \end{array}\right)}_{kp\times kp},$$

Equation (5) can be written as a VAR model of a one-order partitioned matrix, as shown in (7):

(7) $$Z_{t}=A+\Phi Z_{t-1}+E_{t},$$

where the characteristic equation is shown in (8).

(8) |Φ−λI|=0.

For the VAR model, an equivalent condition for the stability was that the solutions of Equation (8) were within the unit circle.

#### 2.2.2. Cointegration Test

The cointegration relationship expresses the long-term dynamic equilibrium relationship between the multiple economic variables interacting with each other and their evolution [56]. Typical cointegration test methods mainly include the Engle-Granger (E-G) two-step method and the Johansen–Juselius (JJ) test method [57,58]. Compared with the EG method, the JJ method cannot only test the multi-cointegration relationship, but also impose constraints on the cointegration relationship, which was therefore employed in this paper.

Assume that the series $Y_{t}={\left(y_{1,t},\;y_{2,t},\;\cdots ,\;y_{k,t}\right)}^{T}$ is an h order integration. If there exists a k dimension vector β, such that $\beta Y_{t}$ is an h−b order integration, i.e., 0≤b≤h, then $Y_{t}={\left(y_{1,t},\;y_{2,t},\;\cdots ,\;y_{k,t}\right)}^{T}$ is an (h, b) order cointegration and β is a cointegration vector.

For instance, when h=1, the series $Y_{t}={\left(y_{1,t},\;y_{2,t},\;\cdots ,\;y_{k,t}\right)}^{T}$ is integrated of order one. After the differential transform, Equation (1) can be rewritten as (9).

(9) $$\Delta Y_{t}=\Omega Y_{t-1}+\sum_{i=1}^{p-1}\Gamma_{i}\Delta Y_{t-i}+HX_{t}+U_{t},$$

where $\Omega =\sum_{i=1}^{p}\Pi_{i}-I$ and $\Gamma_{i}=-\sum_{j=i+1}^{p}\Pi_{j}$. Since $\Delta Y_{t-i}\;\left(i=0,\;1,\;\cdots ,\;p\right)$ is a vector of order zero, whether a cointegration relationship exists or not depends on the rank of Ω, as explained below.

Denote the rank of Ω by r. When r=0, Ω is a null matrix, so there is no cointegration relationship between variables. When r=k, Ω is a full rank matrix, then $Y_{t}$ is a stationary series, which contradicts with hypotheses. When 0<r<k, there exist r cointegration relationships. The matrix Ω can be written as in (10).

(10) $$\Omega =\alpha \beta^{T},$$

where both the ranks of α and β are r. By inserting Equation (10) into Equation (9), it yields:

(11) $$\Delta Y_{t}=\alpha \beta^{T}Y_{t-1}+\sum_{i=1}^{p-1}\Gamma_{i}\Delta Y_{t-i}+HX_{t}+U_{t},$$

where $\beta^{T}Y_{t-1}$ refers to the vector of cointegration relationship. β refers to a matrix of cointegration vectors.

The number of eigenvalues of Ω is usually judged from the trace test and the max-eigenvalue test. The eigenvalues of Ω are $\lambda_{1},\lambda_{2},\cdots ,\lambda_{k}$, where $\lambda_{1}>\lambda_{2}>\cdots >\lambda_{k}$. The null hypothesis was that there existed r cointegration relationships at most; the alternative hypothesis was that there existed r+1 cointegration relationships at least. The statistics of the trace test and max-eigenvalue test are shown in (12) and (13), respectively.

(12) $$\eta_{r}=-T\sum_{i=r+1}^{k}\ln\left(1-\lambda_{i}\right).$$

(13) $$\xi_{r}=-T\ln\left(1-\lambda_{r+1}\right).$$

At significance level α, the values of statistics $\eta_{r}$ and $\xi_{r}$ were compared with the corresponding critical values from r=0. The null hypothesis was accepted if the values of statistics were smaller than the critical values, which meant that there existed r cointegration relationships at most. Otherwise, the null hypothesis was rejected, which meant that there existed r+1 cointegration relationships at least. The order r was increased in turn until the null hypothesis was accepted.

#### 2.2.3. Granger Causality Test

For economic variables, some were highly correlated, but this did not necessarily mean there was a causal relationship between them. The causal relationship was defined as follows [59]: if x is the cause of the change in y, then when regressing y on its past values, additional past values of x will remarkably improve the explanatory power of the regression.

Given a k-dimensional VAR(*p*) model, as shown in (14):

(14) $${\left(\begin{array}{c} y_{1}\\ \vdots \\ y_{k} \end{array}\right)}_{t}=\left(\begin{array}{ccc} \pi_{11}^{\left(1\right)} & \cdots & \pi_{1k}^{\left(1\right)}\\ \vdots & \vdots & \vdots \\ \pi_{k1}^{\left(1\right)} & \cdots & \pi_{kk}^{\left(1\right)} \end{array}\right){\left(\begin{array}{c} y_{1}\\ \vdots \\ y_{k} \end{array}\right)}_{t-1}+\cdots +\left(\begin{array}{ccc} \pi_{11}^{\left(p\right)} & \cdots & \pi_{1k}^{\left(p\right)}\\ \vdots & \vdots & \vdots \\ \pi_{k1}^{\left(p\right)} & \cdots & \pi_{kk}^{\left(p\right)} \end{array}\right){\left(\begin{array}{c} y_{1}\\ \vdots \\ y_{k} \end{array}\right)}_{t-p}+\left(\begin{array}{ccc} h_{11} & \cdots & h_{1d}\\ \vdots & \vdots & \vdots \\ h_{k1} & \cdots & h_{kd} \end{array}\right){\left(\begin{array}{c} x_{1}\\ \vdots \\ x_{d} \end{array}\right)}_{t}+{\left(\begin{array}{c} u_{1}\\ \vdots \\ u_{k} \end{array}\right)}_{t},$$

the variable $y_{j}$ was not the Granger cause of the variable $y_{i}$ if and only if ∀q∈[1,p], q∈N, and it holds that $\pi_{ij}^{\left(q\right)}=0$.

Therefore, the null hypothesis of the test was $\pi_{ij}^{\left(q\right)}=0,\;q=1,\;2,\;\cdots ,\;p$; the alternative hypothesis was that there at least existed a q, such that $\pi_{ij}^{\left(q\right)}\neq 0$. The statistic is written as (15).

(15) $$S=\frac{T\left(SSR_{0}-SSR_{1}\right)}{SSR_{1}},$$

where $SSR_{1}$ is the residual square sum of $y_{i}$ in Equation (14). $SSR_{0}$ is the residual square sum of $y_{i}$ without the lagged variable $y_{j}$. T is the sample number, and p is the lag order.

At significance level α, the value of S was compared with the critical value. When S was smaller than the critical value, the null hypothesis was accepted, which meant that the variable $y_{j}$ was not the Granger cause of $y_{i}$; otherwise, the null hypothesis was rejected (i.e., the variable $y_{j}$ was the Granger cause of variable $y_{i}$.)

### 2.3. Stationary Sequences

In a stationary sequence the statistical law does not change over time. For a time series generated from a random process, $\left\{x_{t}\right\}\left(t=1,\;2,\;\cdots ,\;T\right)$, if the sequence is stationary, the following conditions must be satisfied:

(1) ∀t, $E\left(x_{t}\right)=\mu$;

(2) ∀t, $Var\left(x_{t}\right)=\sigma^{2}$;

(3) ∀t, $Cov\left(x_{t},x_{t+k}\right)=\gamma_{k}$, where $\gamma_{k}$ is only related to k.

In practical applications, the economic and financial sequences encountered are often non-stationary time series. If they are forced to return, they may lead to “false return” problems. Therefore, stationary data needs to be tested before modeling. Stationary sequences were examined by the augmented Dickey–Fuller (ADF) test method.

An ADF test is used to control a higher order correlation by adding the lag difference term of $x_{t}$ on the right side of the regression equation. Consider the following three models:

(16) $$x_{t}=\sum_{i=1}^{p}\varphi_{i}x_{t-i}+u_{t},$$

(17) $$x_{t}=a+\sum_{i=1}^{p}\varphi_{i}x_{t-i}+u_{t},$$

(18) $$x_{t}=a+bt+\sum_{i=1}^{p}\varphi_{i}x_{t-i}+u_{t},$$

where a is a constant term. bt is a linear trend function. $u_{t}$ is a random error term. p is the lag order, which is usually determined by AIC.

Subtract $x_{t-1}$ from both ends of Equations (16)–(18),

(19) $$\Delta x_{t}=\eta x_{t-1}+\sum_{i=1}^{p}\beta_{i}\Delta x_{t-i}+u_{t},$$

(20) $$\Delta x_{t}=a+\eta x_{t-1}+\sum_{i=1}^{p}\beta_{i}\Delta x_{t-i}+u_{t},$$

(21) $$\Delta x_{t}=a+bt+\eta x_{t-1}+\sum_{i=1}^{p}\beta_{i}\Delta x_{t-i}+u_{t},$$

where $\eta =\sum_{i=1}^{p}\varphi_{i}-1$, $\beta_{i}=-\sum_{j=i+1}^{p}\varphi_{j}$.

Model (20) contained intercept items without time trend items, while Model (21) contained both intercept and time trend terms. In practical applications, Model (21) was usually established to test the significance of intercept and trend terms. If they were significant, then they can be retained; otherwise, they should be rejected, and the model can be simplified to the form of (19) or (20).

The null hypothesis of this test was η=0; that is, there existed a unit root in the sequence. The alternative hypothesis was η<0; that is, there existed no unit root. The test statistic is shown in (22).

(22) $$\tau =\frac{\hat{\eta }}{S\left(\hat{\eta }\right)},$$

where $\hat{\eta }$ is the estimate of $\eta ,\;\mathrm{and}\;S\left(\hat{\eta }\right)$ is the standard deviation of $\hat{\eta }$.

At significance level α, if the null hypothesis was not rejected, there was a unit root in the sequence; otherwise, this sequence was stationary.

### 2.4. Impulse Response Function

An impulse response function was employed to study the dynamic effects of disturbance items on the current and future of endogenous variables. It was also employed to analyze the influential relationships between variables, since exogenous variables were not affected by system shocks [60]. Consider the VAR(*p*) model in (1) without exogenous variables, as shown in (23):

(23) $$Y_{t}=\Pi_{1}Y_{t-1}+\Pi_{2}Y_{t-2}+\cdots +\Pi_{p}Y_{t-p}+U_{t},\;t=1,\;2,\;\cdots ,\;T,$$

where T is the sample size. $Y_{t}={\left(y_{1,t},\;y_{2,t},\;\cdots ,\;y_{k,t}\right)}^{T}$ is a k dimensional endogenous variable vector.$\Pi_{1},\;\Pi_{2},\;\cdots ,\;\Pi_{p}$ are k×k estimated coefficient matrices. $U_{t}={\left(u_{1,t},\;u_{2,t},\;\cdots ,\;u_{k,t}\right)}^{T}$ is k dimensional random error vector.

Equation (23) can be rewritten as an infinite-order vector moving average model, as in (24).

(24) $$\begin{array}{ll} Y_{t} & ={\left(I-\Pi_{1}L-\Pi_{2}L^{2}-\cdots -\Pi_{p}L^{p}\right)}^{-1}U_{t}\\ & =\left(\Theta_{0}+\Theta_{1}L+\Theta_{2}L^{2}+\cdots \right)U_{t} \end{array}$$

where L is a casual operator. $\Theta_{i}$ satisfies:

$\left(I-\Pi_{1}L-\Pi_{2}L^{2}-\cdots -\Pi_{p}L^{p}\right)\left(\Theta_{0}+\Theta_{1}L+\Theta_{2}L^{2}+\cdots \right)=I$, $\Theta_{0}=I$.

Equation (24) can be transformed into:

(25) $$Y_{t+s}=\left(I+\Theta_{1}L+\Theta_{2}L^{2}+\cdots \right)U_{t+s}.$$

According to Equation (25), the following holds:

(26) $$\Theta_{s}=\frac{\partial Y_{t+s}}{\partial U_{t}}.$$

Then the element of the i^th^ row and the j^th^ column of $\Theta_{s}$ can be denoted as below:

(27) $$\theta_{ij}=\frac{\partial y_{i,t+s}}{\partial u_{j,t}},\;s=1,\;2,\;\cdots ,$$

where θ is an element of matrix Θ. This indicated that on the condition that other error terms remain unchanged, it would have an impact on endogenous variable $y_{i}$ at time t+s when $u_{j}$ received a shock at time *t*.

However, the covariance matrix was not necessarily a diagonal matrix; that is, the elements in the random error term changed with a certain element, which contradicted with the assumption of the impulse response function. Therefore, by introducing a transformation matrix, the covariance matrix can be converted into a diagonal matrix, so as to achieve the goal of orthogonalizing the error term.

### 2.5. Variance Decomposition

Variance decomposition was used to analyze the contribution of each endogenous variable to the variance decomposition, so as to reveal the relationship between the variables in the system. According to Equation (25), it holds that:

(28) $$y_{i,t}=\sum_{j=1}^{k}\left(\theta_{ij}^{\left(0\right)}u_{j,t}+\theta_{ij}^{\left(1\right)}u_{j,t-1}+\theta_{ij}^{\left(2\right)}u_{j,t-2}+\cdots \right),\;t=1,\;2,\;\cdots ,\;T.$$

Assume that the covariance matrix Σ of $U_{t}$ is a diagonal matrix. By applying $E\left(U_{t}\right)=0$, the variance of $y_{i}$ can be obtained as:

(29) $$\mathrm{var}\left(y_{i}\right)=\sum_{j=1}^{k}\sum_{q=0}^{\infty }{\left(\theta_{ij}^{\left(q\right)}\right)}^{2}\sigma_{jj},\;i=1,\;2,\;\cdots ,\;k,$$

where $\sigma_{jj}$ are the elements of the covariance matrix $\sum^{}$.

In this way, the variance of $y_{i}$ is decomposed into k unrelated effects. Then the relative variance contribution rate is defined as in (30).

(30) $$RVC_{j\to i}\left(\infty \right)=\frac{\sum_{q=0}^{\infty }{\left(\theta_{ij}^{\left(q\right)}\right)}^{2}\sigma_{jj}}{\sum_{j=1}^{k}\sum_{q=0}^{\infty }{\left(\theta_{ij}^{\left(q\right)}\right)}^{2}\sigma_{jj}},\;i,j=1,\;2,\;\cdots ,\;k.$$

The relative variance contribution rate describes the contribution of the disturbance term to variables. The larger $RVC_{j\to i}$ is, the greater the influence of the $j^{th}$ disturbance on the $i^{th}$ variable.

## 3. Empirical Analysis

Results of the empirical analysis are presented in this section, including data sources, implementation of the VAR model, model checking, the impulse response function, and variance decomposition.

### 3.1. Data Sources and Data Manipulation

**Figure 1:**
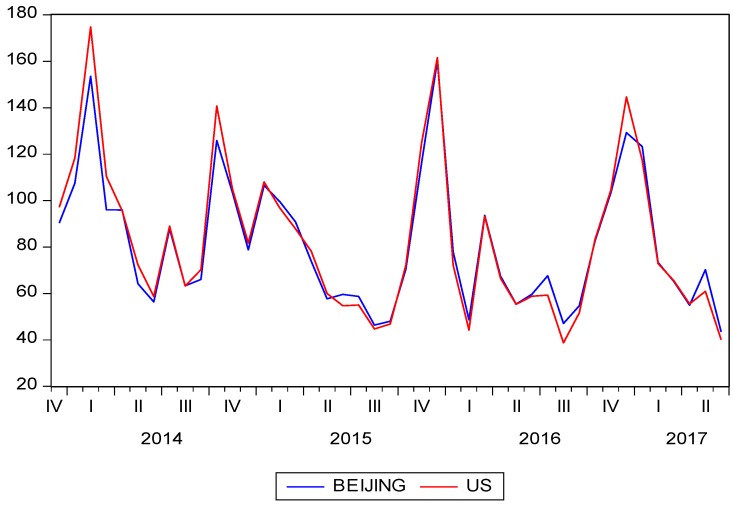
Comparing monthly average PM_2.5_ concentrations between US Embassy monitoring data and 35 monitoring stations in Beijing.

Bidirectional correlation effect between PM_2.5_ pollution and industrial development in Beijing has been analyzed in this paper. In addition to 35 air quality automatic monitoring stations with observational hourly concentration data in Beijing set by the Beijing Environmental Protection Monitoring Center [61], PM_2.5_ data released from the US embassy were also adopted. On one hand, the VAR model constructed in this paper required a longer time span to include a larger number of parameters. The earliest date of the PM_2.5_ data that can be used from the 35 monitoring stations was in December 2013. The US embassy’s monitoring data began in April 2008, which can meet the model needs with a larger sample capacity. On the other hand, the data from the US embassy was as representative as those from 35 monitoring stations. The monthly average PM_2.5_ concentrations between the US Embassy and 35 monitoring stations (from January 2014 to June 2017) was compared in Figure 1. The overall trends for the two sets of observational data were basically the same, with a high correlation coefficient of 0.986 (Figure 1). Liang et al. [62] also compared the two sets of monitoring data, and observed the same result. Therefore, it was reasonable to adopt the US Embassy monitoring data with a longer query period, which was effective and representative.

Statistics on the GDP (unit: 100 million yuan) of the first quarter of 2010 to the second quarter of 2017 can be retrieved from the quarterly statistics released by the Beijing Municipal Bureau of Statistics [63]. Industry development (i.e., economic growth) was measured by the yearly growth rate of gross output by industry (i.e., rate of primary industry (RPI), rate of secondary industry (RSI), and rate of tertiary industry (RTI). The hourly concentration data of PM_2.5_ from January 2010 to June 2017 was collected and converted to quarterly concentration data. This served as the indicator for different haze pollution levels based on the development of RPI, RSI, and RTI. In addition, the monthly consumer price index was obtained from the Beijing Municipal Bureau of Statistics and converted into a quarterly index based on the first quarter of 2010 to eliminate the impact of price fluctuations, according to Equation (31).

(31) $$\frac{currentproductionvalueofyeart}{comparableproductionvalueofyeart}=\frac{consumerpriceindexofyeart}{100}$$

### 3.2. PM_2.5_ and the Trends of the Three Industries

**Figure 2:**
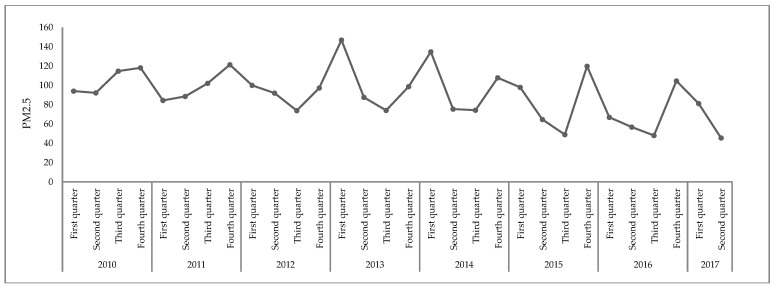
The quarterly variation trends of PM_2.5_ during 2010–2017.

There was a clear seasonal variation of PM_2.5_ pollution, where the most serious pollution appeared in winter (Figure 2). The main reason was because the bad meteorological conditions impeded the diffusion and dilution of pollutants. During the winter season, water vapor in the air was prone to super-saturation, and it condensed into fog because of the large difference between day and night temperatures, relative high humidity, and static wind.

**Figure 3:**
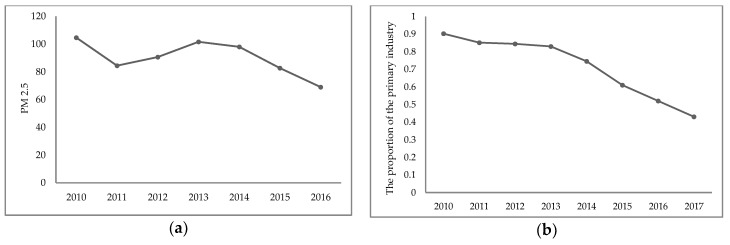

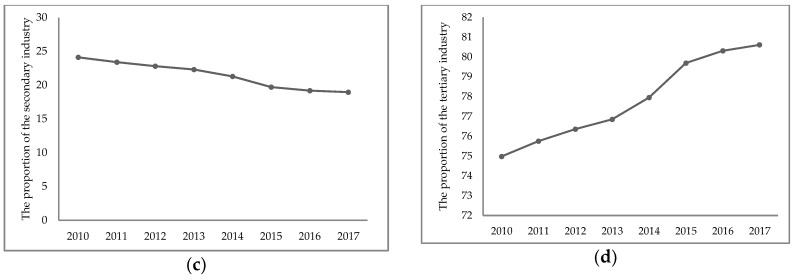
PM_2.5_ concentration and annual changes in the proportion of the three industries. (**a**) Annual changes of PM_2.5_ concentration. (**b**) Annual changes in the proportion of the primary industry GDP. (**c**) Annual changes in the proportion of the secondary industry GDP. (**d**) Annual changes in the proportion of the tertiary industry GDP.

The annual average concentration of PM_2.5_ in Beijing showed a general downward trend (Figure 3a). With the adjustment of industry structures, the output values of the primary, secondary, and tertiary industry also changed. The proportion of the output value of primary industry sharply decreased from 0.9% in 2010 to 0.43% in 2017 (Figure 3b). The proportion of secondary industry gradually dropped from 24.12% in 2010 to 18.97% in 2017 (Figure 3c). On the contrary, the proportion of tertiary industry increased from 74.98% in 2010 to 80.60% in 2017 (Figure 3d). The structural ratios of the three industry output values were 0.43%, 18.97%, and 80.60%, respectively.

### 3.3. Data Test

**Figure 4:**
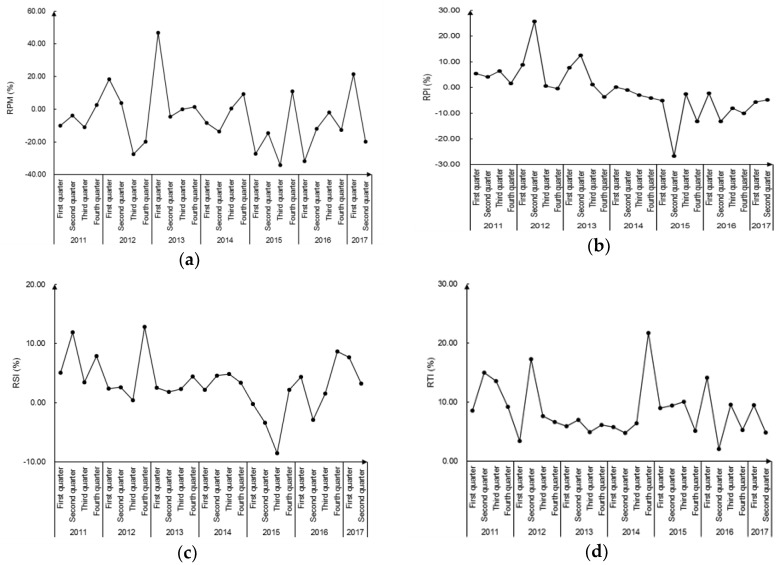
Time series of the variables. (**a**) Changes in the yearly growth rate of PM_2.5_ concentration. (**b**) Changes in the yearly growth rate of the primary industry GDP. (**c**) Changes in the yearly growth rate of the secondary industry GDP. (**d**) Changes in the yearly growth rate of the tertiary industry GDP.

The yearly growth rate of PM_2.5_ concentration was abbreviated as RPM. The yearly growth rates of the primary, secondary, and tertiary industry production were respectively denoted by RPI, RSI, and RTI. The time series variation of each variable is drawn in Figure 4.

The seasonal effects of the variables were firstly tested through the stable seasonality test and the parameter-shift seasonality test using the X-12-ARIMA seasonal adjustment method. According to the results shown in Table 1, none of the four series had a stable seasonality nor a moving seasonality.

In order to avoid the problem of false return, an ADF test was then adopted to examine the smoothness of the time series data. Models (20)–(22) were established for each of the four sequences in turn, to examine the significance of the trend item and the intercept item, so the stationary type could be determined. According to the specific test results shown in Table 2, the unit root test of *RPM*, *RPI*, *RSI,* and *RTI* sequences rejected the null hypothesis. Thus, it was able to consider that all four sequences were stationary sequences, and a VAR model for direct analysis could be established.

### 3.4. Establishing the VAR Model

Before establishing a VAR model, it was required to determine the optimal lag order of the model based on criteria such as LR statistics, FPE, AIC, SC, and HQ. The optimal lag order was four for the FPE, AIC, and HQ, one for the LR, and zero for the SC, respectively (in Table 3). Considering the validity of the model construction, the lag order was set to four for the VAR model, so a VAR(4) model for RPM, RPI, RSI, and RTI was established. As the VAR model was a non-theoretical model, its coefficients had no economic significance. When the VAR model was analyzed, the magnitudes or the positive and negative influences of one variable on another were not emphasized. Therefore, the significance of the coefficients was not analyzed here.

### 3.5. Checking the Model

**Figure 5:**
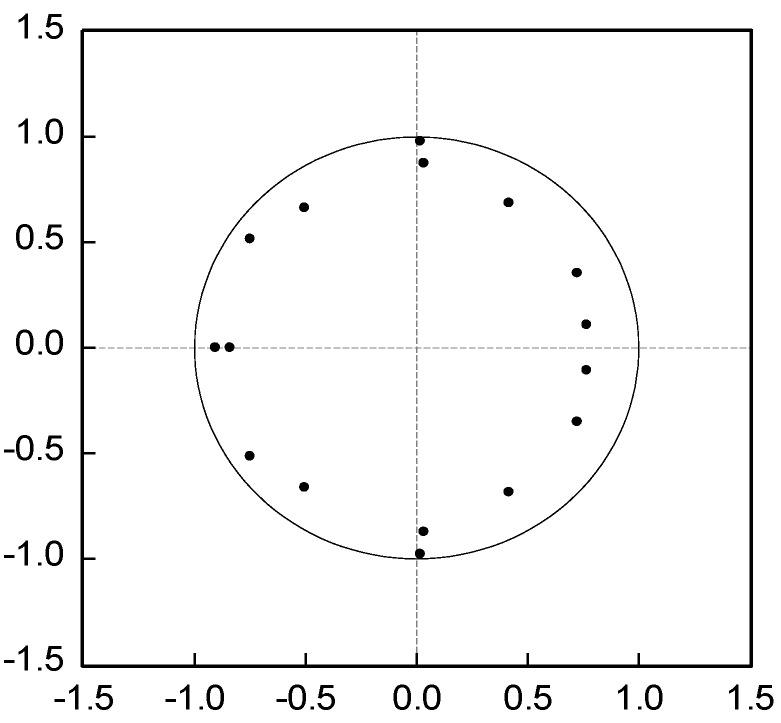
Unit root distribution map.

The stability of the model by the unit root diagram of the VAR model is examined in Figure 5. The unit roots of the model all fell within the unit circle, indicating that Model (33) was stationary and effective [64].

(32) $${\left[\begin{array}{c} RPM\\ RPI\\ RSI\\ RTI \end{array}\right]}_{t}=\left[\begin{array}{c} -0.142\\ -0.117\\ 0.089\\ 0.102 \end{array}\right]+\left[\begin{array}{cccc} -0.220 & -0.237 & 2.587 & -1.867\\ 0.130 & -0.158 & 0.520 & -0.522\\ -0.005 & 0.058 & 0.213 & -0.315\\ -0.038 & 0.085 & -0.189 & -0.151 \end{array}\right]{\left[\begin{array}{c} RPM\\ RPI\\ RSI\\ RTI \end{array}\right]}_{t-1}\;+\left[\begin{array}{cccc} -0.483 & -0.852 & -0.551 & -0.121\\ -0.020 & 0.521 & 0.283 & -0.545\\ -0.155 & 0.195 & -0.242 & -0.134\\ -0.158 & 0.138 & 0.275 & -0.111 \end{array}\right]{\left[\begin{array}{c} RPM\\ RPI\\ RSI\\ RTI \end{array}\right]}_{t-2}\;+\left[\begin{array}{cccc} -0.220 & 1.949 & 2.327 & -1.606\\ -0.095 & 0.177 & 0.170 & 0.749\\ -0.108 & 0.088 & 0.375 & -0.752\\ -0.128 & -0.185 & 0.015 & -0.117 \end{array}\right]{\left[\begin{array}{c} RPM\\ RPI\\ RSI\\ RTI \end{array}\right]}_{t-3}\;+\left[\begin{array}{cccc} -0.468 & -0.406 & 0.174 & 2.139\\ -0.035 & 0.048 & 0.075 & 1.029\\ 0.025 & -0.228 & 0.236 & 0.164\\ -0.031 & -0.030 & 0.662 & -0.325 \end{array}\right]{\left[\begin{array}{c} RPM\\ RPI\\ RSI\\ RTI \end{array}\right]}_{t-4}+\left[\begin{array}{c} {\hat{\varepsilon }}_{1}\\ {\hat{\varepsilon }}_{2}\\ {\hat{\varepsilon }}_{3}\\ {\hat{\varepsilon }}_{4} \end{array}\right]$$

Since each variable examined by the ADF test was a zero-order integrated sequence, as shown in Table 2, the JJ cointegration tests were performed. According to the results shown in Table 4, two cointegration relationships between variables in the model were found at the 5% significance level, which implied a long-term equilibrium relationship between PM_2.5_ pollution and the economic growth of the three industries.

The first cointegration relationship is standardized as:

(33) RPM=0.6888RPI+1.1622RSI−1.3622RTI+0.0051T,

where *T* is a trend variable.

According to Equation (33), the PM_2.5_ concentration increased with the economic growth of the primary and secondary industries; whilst the economic growth of the tertiary industry had an inhibitory effect on PM_2.5_ emissions. The tertiary industry had the greatest impact on PM_2.5_ among the three industries, whereas the primary industry had the least.

Through the Granger causality test, a causal relationship between two variables was further considered, as shown in Table 5; that is, all three industries had a one-way Granger causal relationship with PM_2.5_ at the 5% significance level. There existed a one-way Granger cause from three industries to PM_2.5_, which suggested the development of the three industries in Beijing had a significant impact on PM_2.5_ pollution.

### 3.6. Impulse Response Function

**Figure 6:**
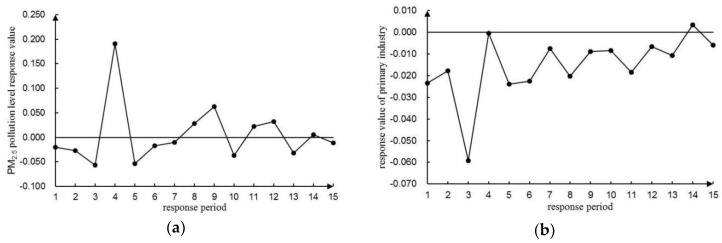
Impulse response function diagram of PM_2.5_ pollution level and development level of the primary industry. (**a**) Impulse response function diagram of the PM_2.5_ pollution level to the primary industry development level. (**b**) Impulse response function diagram of the primary industry development level to the PM_2.5_ pollution level.

**Figure 7:**
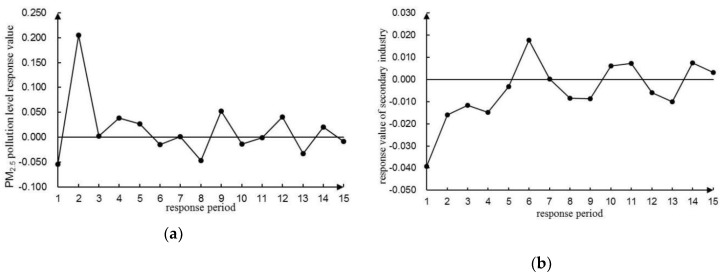
Impulse response function diagram of PM_2.5_ pollution level and development level of the secondary industry. (**a**) Impulse response function diagram of the PM_2.5_ pollution level to the secondary industry development level. (**b**) Impulse response function diagram of the secondary industry development level to the PM_2.5_ pollution level.

**Figure 8:**
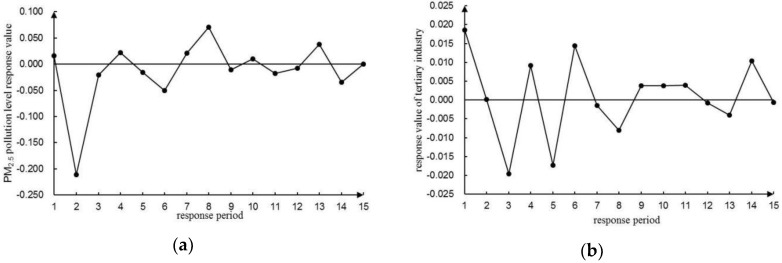
Impulse response function diagram of PM_2.5_ pollution level and development level of the tertiary industry. (**a**) Impulse response function diagram of the PM_2.5_ pollution level to the tertiary industry development level. (**b**) Impulse response function diagram of the tertiary industry development level to the PM_2.5_ pollution level.

To avoid the order effect of variables on the results of the VAR(4) model, a lag period of 15 (i.e., a quarter) was selected using a generalized impulse response function [65,66] for analyzing the dynamic interconnections of PM_2.5_ pollution to the development of the three industries. In Figure 6, Figure 7 and Figure 8, the trajectory of the shock response was analyzed and illustrated between the level of PM_2.5_ pollution and the development of three industries.

#### 3.6.1. Dynamic Relationship between PM_2.5_ Pollution and the Development of Primary Industry

According to Figure 6a, the impact of PM_2.5_ on the primary industry was rather complex, which had both positive and negative effects. PM_2.5_ began to decline in the first period, and dropped to the lowest point of −0.057 in the third period. Then it rose sharply to the peak value of 0.191 and quickly fell back to a very low point. After that, positive effects were depicted in the 8th and 9th periods, but they turned back to negative again in the 10th period. In the final stage, the trend followed fluctuations around zero with a cumulative value of 0.075, which indicated that the development of the primary industry had a weak effect on PM_2.5_ pollution because the source of PM_2.5_ emissions from the primary industry was very limited. Besides, the primary industry accounted for a rather small proportion of the total industry (only 0.43% by 2017). Thus, the development of the primary industry eventually played a minor role resulting in PM_2.5_ pollution.

According to Figure 6b, the response value of the primary industry to PM_2.5_ shocks was mainly negative after a positive impact by PM_2.5_. The primary industry fell to −0.023 in the first period, then rose to −0.018 in the second period. It again sharply dropped to the lowest point of −0.059 in the third period, and increased again to −0.0004 in the fourth period. There was also a gradual fluctuation around zero with a cumulative value of −0.230, which suggested that PM_2.5_ pollution had a certain inhibitory effect on the economic growth of the primary industry. Research in the field of agriculture has demonstrated that high levels of PM_2.5_ can lead to crop yield loss and quality reduction [7].

#### 3.6.2. Dynamic Relationship between PM_2.5_ Pollution Level and the Development of Secondary Industry

According to Figure 7a, influences of PM_2.5_ on the secondary industry were generally positive. The response value of PM_2.5_ dropped to −0.055 in the first period, then it rapidly increased to the peak value of 0.205 in the second period. It decreased to 0.003 in the third period, and then rose again to 0.038 in the fourth period. After that, the response value began to drop to −0.047 again in the eighth period. This repeated negative effect depicted that the continuous action of energy conservation and of industrial structure optimization in the secondary industry was assumed. Finally, the response value kept fluctuating around zero with a cumulative value of 0.215, indicating that the economic growth of the secondary industry exacerbated PM_2.5_ pollution to a certain extent. Although the proportion of the secondary industry to total industries dropped to 18.97% by 2017, it was still the largest source of PM_2.5_. The strong effect was concentrated on several sectors, such as secondary industry pollutants, exhaust emissions, and construction dust.

The response value of PM_2.5_ to the impact of the second industry was all negative in the first five periods, shown in Figure 7b. The lowest point of −0.039 appeared in the first period, and reached the peak value of 0.018 in the sixth period. Then it fluctuated around zero with a cumulative value of −0.075, which demonstrated that PM_2.5_ pollution hindered the economic growth of the second industry in a short term. Such a short-term response can be explained as temporarily limited or ceased production of relevant emission enterprises.

#### 3.6.3. Dynamic Relationship between PM_2.5_ Pollution and the Development of Tertiary Industry

According to Figure 8a, the response of PM_2.5_ to the impact of the tertiary industry was mainly inhibited. The response value of PM_2.5_ rose to 0.017 in the first period, fell to the lowest value of −0.211 in the second period, and rose to −0.020 in the third period. After the fourth period it experienced a temporary positive effect, and gradually stabilized at zero over time with a cumulative value of −0.189, which shows=ed that the development of the tertiary industry alleviated the PM_2.5_ pollution to some extent. Meanwhile, the temporary positive effect demonstrated that some sectors promoted PM_2.5_ emissions, for example, the motor vehicle exhaust emissions and dust emission in transportation, postal trades, etc.

From Figure 8b, it was found that the response of PM_2.5_ to the tertiary industry alternated between positive and negative impacts. The tertiary industry reached a peak at 0.019 in the first period and fell to the lowest point of −0.020 in the third period. It then rose to 0.009 in the fourth period. After that, it gradually fluctuated around zero with a cumulative value of the first five periods of −0.009. It indicated that in the short term, PM_2.5_ pollution had a certain inhibitory effect on the tertiary industry, but it was not significant and had a weak driving effect on the tertiary industry in the long term.

### 3.7. Variance Decomposition

Variance decomposition was performed in order to analyze the degree of mutual contribution between the development level of the three industries and the level of PM_2.5_ pollution based on the VAR(4) model. Therefore, a lag period of 5 was chosen. The results of the Cholesky decomposition method were very sensitive to the order of variables, and thus this method limited the causal order of variables over the same period. Therefore, based on the research purpose, the corresponding economic theory, as well as the results of the Granger causality test in Table 5, we determined the order of variables as RTI > RSI > RPI > RPM, as shown in Table 6.

According to the variance decomposition result of PM_2.5_, the contribution rate of the primary industry gradually increased and peaked at 37% in the fifth period, and then gradually stabilized to 34%. The contribution rate of the secondary industry gradually decreased from 66% and stabilized at 18%. The contribution of the tertiary industry reached the peak of 73% in the second period, then gradually decreased, and tended to stabilize. It was apparent that the impact of the primary and tertiary industrial developments had a certain lag effect on PM_2.5_ emissions. The secondary industry was responsible for PM_2.5_ emissions from the very beginning, since it contained almost all the high energy consumption industries that led to heavy pollution. However, its role in raising PM_2.5_ gradually weakened. Thus, the main factor affecting the level of PM_2.5_ pollution was the secondary industry in the early period, and then it turned to the primary and tertiary industries for the rest of the periods, as shown in Table 6.

The impact of PM_2.5_ in the first period was zero, demonstrating that PM_2.5_ pollution had a lagging effect on the development of the three industries. This result was quite in line with the real practice of “treatment after pollution”. The contribution rate of PM_2.5_ pollution to the secondary industry was the largest, reaching 1.6% in the 8th period. The contribution rate to the tertiary industry was only 0.4%, whereas to the primary industry is was less than 0.2% for the same period. Hence, the boost in secondary industry in Beijing was most sensitive to PM_2.5_. This observation was rational since the secondary industry mainly influenced air pollution.

## 4. Discussion

The impulse response function indicated that economic growth of the primary and secondary industries can raise PM_2.5_ emissions, whilst the tertiary industry had an inhibitory effect. This result is consistent with the findings of Cao [67] and Zhao [68]. Therefore, it can be found that industry improvement (defined as economic growth) is an important factor affecting the intensity and direction of PM_2.5_ emissions. More efforts should be undertaken to implement innovation-driven development strategies, promote industry transformation and upgrade, vigorously develop the tertiary industry, and actively cultivate high-end service industries in order to mitigate PM_2.5_ pollution.

In addition, PM_2.5_ pollution itself can hinder the development of the primary and secondary industries. In the long run, it can promote the development of the tertiary industry, which reveals that PM_2.5_ pollution can influence industry growth as well. With the intensification of pollution and the enhancement of environmental awareness, the advancement of tertiary industry would become one of the most effective ways to alleviate PM_2.5_ pollution.

Liu et al. [48], Duan et al. [52], and Li et al. [69] all observed that indicators of economic growth had good predictions of environmental pollution, while indicators of environmental pollution had less ability to interpret the variance of economic growth. Results indicated by variance decomposition is supported by their studies, which shows that the industry development of Beijing leans over the cost of PM_2.5_ pollution. Capital and labor input are important driving forces for the improvement of Beijing’s industries, whereas the impact of PM_2.5_ pollution is relatively small. Environmental quality preference will also restrict the adverse effects of PM_2.5_ pollution on industry growth. Therefore, while the industry is developing rapidly, it is necessary to pay attention to alleviating the negative effects of environmental pollution.

Nonetheless, results in this paper were based on statistical characteristics of the data, which were limited by the characteristics of the VAR model, and were not closely integrated with related economic theories. Additionally, several impact factors of industry development and PM_2.5_ pollution, such as policy environment and environmental quality preference, are not considered in this paper. Therefore, the explanation of impact mechanisms in this paper can be further investigated with in-depth data mining. Overall, conclusions drawn in this paper emphasized the economic losses caused by PM_2.5_ as well as the interaction of PM_2.5_ with industry development, which cannot be overlooked.

## 5. Conclusions

From the perspective of industry development, a VAR model has been proposed in this paper to examine the dynamic relation between the level of haze pollution and industry progress in 2010–2017 through an impulse response function and a variance decomposition method. Results have shown that a long-term equilibrium relationship existed between PM_2.5_ pollution and the improvement of the primary, secondary, and tertiary industries.

The three industries are the one-way Granger causes of PM_2.5_. The development of the three industries have shown different degrees of impact on the PM_2.5_ pollution, with the duration of about one to two years. The development of the primary and secondary industries would increase PM_2.5_ emission, whilst the tertiary industry would reduce.

The research work in this paper focuses on analyzing the two-way mechanism between PM_2.5_ pollution and the three industries from the perspective of time dynamics, so as to provide theoretical support for Beijing’s industry development and environmental planning. The foundation of haze management is to adjust industrial development to promote industry transformation and upgrade. Emergency measures, such as suspending the production of heavy polluting enterprises and restricting motor vehicle operation in the city, can only alleviate heavy pollution in the short-term. The long-term solution to the problem of haze requires transforming the extensive economic development model, optimizing the industry structure, vigorously developing the tertiary industry, and forcing the transformation and upgrade enterprises so as to achieve coordinated economic and environmental development.

## Figures and Tables

**Table 1:** Seasonal Test Results.

Variables	Stable Seasonality Test		Moving Seasonality Test	
	F statistics	Conclusion	F statistics	Conclusion
RPM	1.271	no evidence of stable seasonality	0.600	no evidence of moving seasonality
RPI	1.258	no evidence of stable seasonality	1.321	no evidence of moving seasonality
RSI	6.197	no evidence of stable seasonality	1.070	no evidence of moving seasonality
RTI	0.060	no evidence of stable seasonality	0.771	no evidence of moving seasonality

RPI: rate of primary industry, RSI: rate of secondary industry, RTI: rate of tertiary industry. RPM: The yearly growth rate of PM_2.5_ concentration was abbreviated as RPM.

**Table 2:** Augmented Dickey–Fuller (ADF) Test Results.

Variables	Test Form	ADF Statistics	Stationarity	Trend Item	Intercept	Lag Order	Conclusion
RPM	(C,T,K)	−5.786205	stationary **	none	none	0	stationary without intercept item and trend item
	(C,0,K)	−5.671215	stationary **	—	none	0	
	(0,0,K)	−5.292310	stationary **	—	—	0	
RPI	(C,T,K)	−4.835591	stationary **	existence **	existence **	0	stationary with trend item
	(C,0,K)	—	—	—	—	—	
	(0,0,K)	—	—	—	—	—	
RSI	(C,T,K)	−3.960725	stationary **	none	existence *	0	stationary with intercept item
	(C,0,K)	−3.804746	stationary **	—	existence **	—	
	(0,0,K)	—	—	—	—	—	
RTI	(C,T,K)	−5.534724	stationary **	none	existence **	0	stationary with intercept item
	(C,0,K)	−5.426812	stationary **	—	existence **	0	
	(0,0,K)	—	—	—	—	—	

“C”, “T”, and “K” represent constant term, time trend, and lag order, respectively; “*” and “**”: reject the null hypothesis at the 10% and 5% significance level, respectively.

**Table 3:** Lag Order.

Lag	LR	FPE	AIC	SC	HQ
0	NA	1.52 × 10^−9^	−8.95534	−8.756966 *	−8.90861
1	29.32872 *	1.19 × 10^−9^	−9.22601	−8.23415	−8.99236
2	10.57243	2.69 × 10^−9^	−8.58473	−6.79939	−8.16416
3	14.85528	3.66 × 10^−9^	−8.78077	−6.20194	−8.17328
4	23.30377	5.60 × 10^−10^ *	−11.98698 *	−8.61467	−11.19256 *

“*”: Represents the selected lag order. LR: likelihood ratio test statistic, FPE: final prediction error, AIC:Akaike information criterion, SC: Schwarz information criterion, HQ: Hannan-Quinn information criterion.

**Table 4:** Johansen–Juselius (JJ) Cointegration Test Results.

Null Hypothesis	Eigenvalue	Trace Test		Maximum Eigenvalue Test	
		Statistics	5% Critical Value	Statistics	5% Critical Value
no cointegration relationship *	0.9784	151.8784	63.8761	84.3802	32.1183
at most one cointegration relationship *	0.9225	67.4982	42.9153	56.2640	25.8232
at most two cointegration relationships	0.2896	11.2342	25.8721	7.5235	19.3870

“*”: Reject the null hypothesis at the 5% significance level.

**Table 5:** Granger causality test results.

Null Hypothesis	χ^2^ Statistics	*p-*Value	Conclusion
RPI is not a Granger cause of RPM	25.55	0.000	refuse *
RSI is not a Granger cause of RPM	37.11	0.000	refuse *
RTI is not a Granger cause of RPM	50.12	0.000	refuse *
All are not a Granger cause of RPM	161.93	0.000	refuse *
RPM is not a Granger cause of RPI	0.94	0.919	accept
RPM is not a Granger cause of RSI	2.76	0.599	accept
RPM is not a Granger cause of RTI	0.80	0.938	accept

“*”: Reject the null hypothesis at the 5% significance level.

**Table 6:** The variance decomposition of three industries and PM_2.5._

Time	Contribution Rate of Three Industries to PM_2.5_			Contribution Rate of PM_2.5_ to Three Industries		
	RPI (%)	RSI (%)	RTI (%)	RPI (%)	RSI (%)	RTI (%)
1	1.4343	66.3280	6.1932	0.0000	0.0000	0.0000
2	0.5011	24.8786	72.6510	0.2118	0.0008	0.0296
3	4.8225	23.6702	69.3508	0.1297	0.6037	0.3569
4	35.3334	18.4177	44.8152	0.1374	0.4304	0.3506
5	37.3774	18.1013	43.0945	0.1152	1.1778	0.3353
6	35.7270	19.3533	43.3784	0.1133	1.2785	0.3639
7	35.7366	19.3414	43.3107	0.1189	1.5279	0.3737
8	34.3263	18.5498	45.5829	0.1519	1.6385	0.3723
